# Supplementary material for: Kinetics and mechanisms of catalyzed dual-E (antithetic) controllers
Source: PLoS One. 2022 Aug 18;17(8):e0262371. doi: 10.1371/journal.pone.0262371 (PMC9387869; doi:10.1371/journal.pone.0262371)
Supplement: S1 Text — (PDF) [file pone.0262371.s001.pdf]

Supporting Material, File S1 Text

Kinetics and mechanisms of catalyzed dual-E (antithetic) controllers

Qaiser Waheed[◐], Huimin Zhou[◐], P. Ruoff[*]
Department of Chemistry, Bioscience, and Environmental Engineering,
University of Stavanger, Norway

[*]Corresponding author. Address: Department of Chemistry, Bioscience, and Environmental Engineering, University of Stavanger, Stavanger, Norway, Tel.: (47) 5183-1887, E-mail: peter.ruoff@uis.no

[◐]Authors contributed equally to this work.

Steady state expressions for the reaction velocities in enzyme-catalyzed removals of E_1 and E_2

We give here three examples how the steady state expressions of v for the enzyme (Ez) catalyzed removals of E_1 and E_2

$$E_1 + E_2 \xrightarrow{\text{Ez}} P \tag{S1}$$

can be derived by using the King-Altman approach (1, 2). In the three examples the enzyme Ez follows random-order or compulsory-order ternary-complex mechanisms, or a ping-pong (substitution) mechanism.

Random-order ternary-complex mechanism

To get the steady-state expression of v by the King-Altman approach we arrange the four enzymatic species as a square:

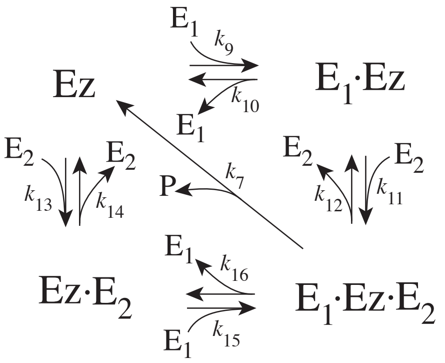

Figure S1. King-Altman arrangement of the enzymatic species in the random-order ternary-complex mechanism converting E_1 and E_2 to P.

Since the $(E_1 \cdot Ez \cdot E_2)$ and Ez corners are connected by the reaction forming P we have to consider the following interconversion patterns (2)

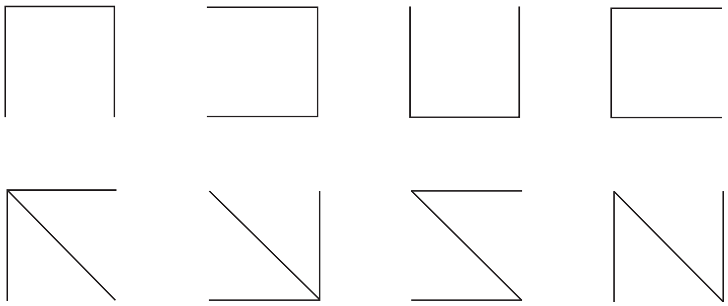

Figure S2. The eight interconversion patterns of the random ternary-complex mechanism that need to be considered.

For the $\mathrm{Ez}/\mathrm{Ez_{tot}}$ ratio we get:

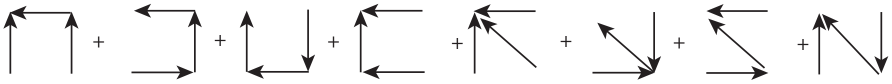

$$\frac{(\mathrm{Ez})}{\mathrm{Ez_{tot}}} = \frac{\cdots}{D}$$

Identifying the contributions of the arrows for each enzyme species leads to

$$\begin{aligned}\frac{(\mathrm{Ez})}{\mathrm{Ez_{tot}}} &= \frac{k_{14}k_{10}k_{12} + k_{15}\mathrm{E}_1k_{12}k_{10} + k_{11}\mathrm{E}_2k_{16}k_{14} + k_{16}k_{14}k_{10}}{D} \\ &+ \frac{k_{14}k_7k_{10} + k_7k_{15}\mathrm{E}_1k_{11}\mathrm{E}_2 + k_{15}\mathrm{E}_1k_7k_{10} + k_{14}k_7k_{11}\mathrm{E}_2}{D}\end{aligned} \tag{S2}$$

For the $(\mathrm{E}_1 \cdot \mathrm{Ez})/\mathrm{Ez_{tot}}$ ratio we have the following pattern contributions:

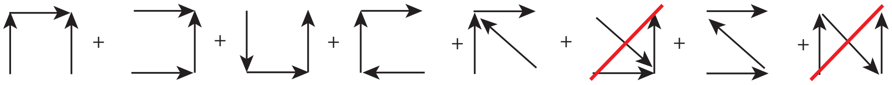

$$\frac{(\mathrm{E}_1{\cdot}\mathrm{Ez})}{\mathrm{Ez_{tot}}} = \frac{\cdots}{D}$$

The red strike-outs indicate reactions that are not considered, since the formation of P from the ternary complex is assumed to be irreversible. Identifying the rates from the arrows gives:

$$\begin{aligned}\frac{(\mathrm{E}_1{\cdot}\mathrm{Ez})}{\mathrm{Ez_{tot}}} &= \frac{k_{14}k_9\mathrm{E}_1k_{12} + k_9\mathrm{E}_1k_{12}k_{15}\mathrm{E}_1 + k_{13}\mathrm{E}_2k_{15}\mathrm{E}_1k_{12}}{D} \\ &+ \frac{k_{16}k_{14}k_9\mathrm{E}_1 + k_{14}k_7k_9\mathrm{E}_1 + k_{15}\mathrm{E}_1k_7k_9\mathrm{E}_1}{D}\end{aligned} \tag{S3}$$

For the $(E_1 \cdot Ez \cdot E_2)/Ez_{tot}$ ratio we get:

$$\frac{(E_1{\cdot}Ez{\cdot}E_2)}{Ez_{tot}} =$$

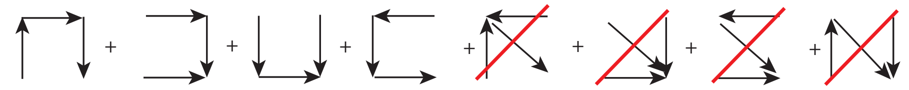

$$D$$

Identifying the rates from the valid patterns gives:

$$\frac{(E_1{\cdot}Ez{\cdot}E_2)}{Ez_{tot}} = \frac{k_{14}k_9 E_1 k_{11} E_2 + k_9 E_1 k_{11} E_2 k_{15} E_1 + k_{10} k_{13} E_2 k_{15} E_1}{D} + \frac{k_{13} E_2 k_{15} E_1 k_{11} E_2}{D} \tag{S4}$$

Finally for the $(Ez \cdot E_2)/Ez_{tot}$ ratio we get:

$$\frac{(Ez{\cdot}E_2)}{Ez_{tot}} =$$

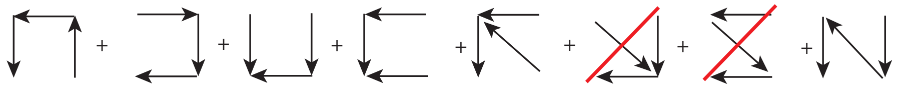

$$D$$

with the corresponding equation:

$$\frac{(Ez{\cdot}E_2)}{Ez_{tot}} = \frac{k_{13} E_2 k_{10} k_{12} + k_9 E_1 k_{11} E_2 k_{16} + k_{13} E_2 k_{16} k_{11} E_2 + k_{10} k_{13} E_2 k_{16}}{D} + \frac{k_{13} E_2 k_7 k_{10} + k_{13} E_2 k_7 k_{11} E_2}{D} \tag{S5}$$

The denominator D is the sum off all 24 numerator terms. The reaction velocity is then written as:

$$v = k_7 (E_1 \cdot Ez \cdot E_2) = V_{max} \left(\frac{k_{14}k_9 E_1 k_{11} E_2 + k_9 E_1 k_{11} E_2 k_{15} E_1 + k_{10} k_{13} E_2 k_{15} E_1 + k_{13} E_2 k_{15} E_1 k_{11} E_2}{D} \right) \tag{S6}$$

where $V_{max} = k_7 (Ez_{tot})$.

Compulsory-order ternary-complex mechanism

In this example E_2 binds first to free enzyme Ez. The arrangement of the enzyme species is triangular:

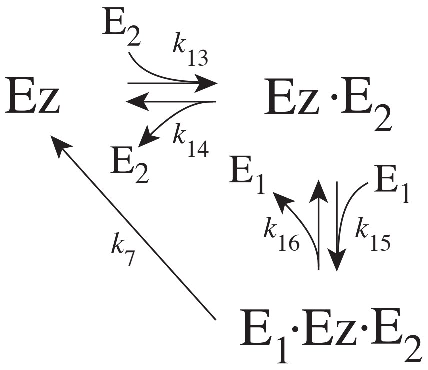

Figure S3. King-Altman arrangement of the enzymatic species in the compulsory-order ternary-complex mechanism when E_2 binds first to Ez.

For the $\mathrm{Ez}/\mathrm{Ez}_{\mathrm{tot}}$ ratio we get:

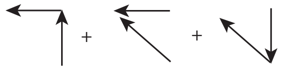

$$\frac{(\mathrm{Ez})}{\mathrm{Ez}_{\mathrm{tot}}} = \frac{\;}{D} = \frac{k_{14}k_{16} + k_7k_{14} + k_7k_{15}\mathrm{E}_1}{D}$$

For the $\mathrm{EzE}_2/\mathrm{Ez}_{\mathrm{tot}}$ ratio we have:

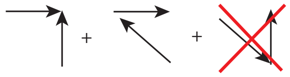

$$\frac{(\mathrm{EzE}_2)}{\mathrm{Ez}_{\mathrm{tot}}} = \frac{\;}{D} = \frac{k_{13}k_{16}\mathrm{E}_2 + k_7k_{13}\mathrm{E}_2}{D}$$

Finally, for the $(\mathrm{E}_1\mathrm{EzE}_2)/\mathrm{Ez}_{\mathrm{tot}}$ ratio we get:

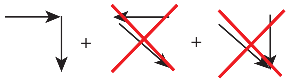

$$\frac{(\mathrm{E}_1\mathrm{EzE}_2)}{\mathrm{Ez}_{\mathrm{tot}}} = \frac{\;}{D} = \frac{k_{13}k_{15}\mathrm{E}_1\mathrm{E}_2}{D} \qquad \text{(S7)}$$

D is the sum of all numerator contributions, i.e.

$$D = k_{13}k_{15}\mathrm{E}_1\mathrm{E}_2 + k_{13}k_{16}\mathrm{E}_2 + k_7k_{13}\mathrm{E}_2 + k_{14}k_{16} + k_7k_{14} + k_7k_{15}\mathrm{E}_1 \qquad \text{(S8)}$$

The velocity v is given by v=$k_7(\mathrm{E}_1\mathrm{EzE}_2)$. Using $(\mathrm{E}_1\mathrm{EzE}_2)$ from Eq S7 we get:

$$v = k_7(\mathrm{E}_1\mathrm{EzE}_2) = V_{max}\frac{k_{13}k_{15}(E_1)(E_2)}{D} \qquad \text{(S9)}$$

with $\mathrm{V}_{\mathrm{max}}$=$\mathrm{k}_7(\mathrm{Ez}_{\mathrm{tot}})$.

Ping-pong mechanism

The considered ping-pong mechanism is shown in Fig S4

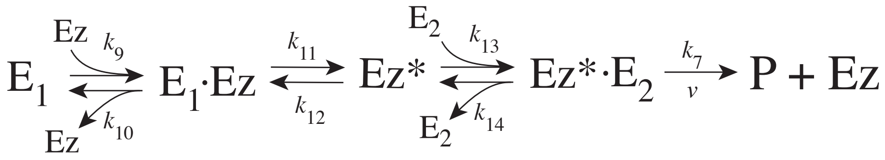

Figure S4. Enzyme Ez uses a ping-pong mechanism in catalyzing the reaction S1.

We assume that E_1 binds to the enzyme Ez first and creates the alternative form Ez* and may release a first product (not considered here). Ez* binds then E_2 and via the intermediate $\mathrm{Ez}^*\cdot\mathrm{E}_2$ the final product P is formed thereby recycling Ez. The reaction velocity v is given by the expression

$$v = k_7(\mathrm{Ez}^*\cdot\mathrm{E}_2) \qquad \text{(S10)}$$

To get the steady-state expression of v, we use the King-Altman approach, by arranging the four enzymatic species as a square:

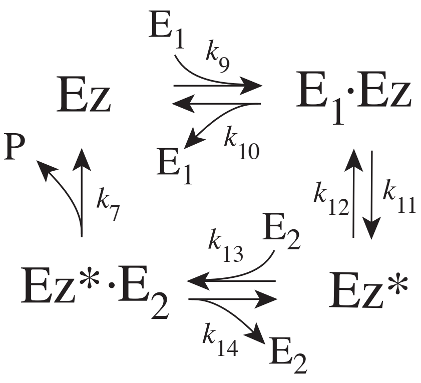

Figure S5. Arrangement of the four enzymatic species in the ping-pong mechanism.

The square arrangement of the enzymatic species gives four interconversion patterns

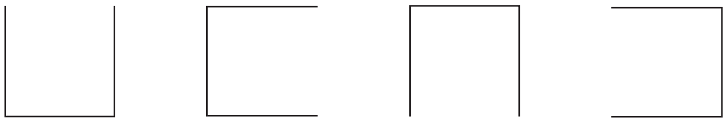

Figure S6. The four interconversion patterns of the ping-pong mechanism.

which are used to calculate the ratios between the different enzymatic species and the total concentration of the enzyme.

For the Ez/ $\mathrm{Ez_{tot}}$ ratio we get:

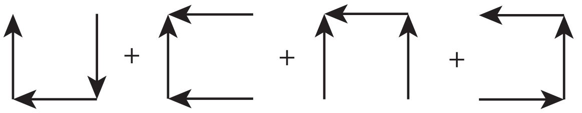

$$\frac{(\mathrm{Ez})}{\mathrm{Ez_{tot}}} = \frac{\qquad}{D}$$

Then, identifying the contributions for each of the arrows leads to

$$\frac{(\mathrm{Ez})}{\mathrm{Ez_{tot}}} = \frac{k_{11}k_{13}\mathrm{E}_2k_7 + k_{13}\mathrm{E}_2k_7k_{10} + k_7k_{10}k_{12} + k_{14}k_{12}k_{10}}{D} \tag{S11}$$

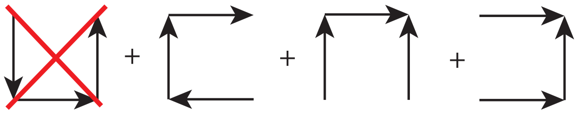

$$\frac{(\mathrm{E_1Ez})}{\mathrm{Ez_{tot}}} = \frac{\qquad}{D} = \frac{k_{13}\mathrm{E}_2k_7k_9\mathrm{E}_1 + k_7k_9\mathrm{E}_1k_{12} + k_{14}k_{12}k_9\mathrm{E}_1}{D} \qquad \text{(S12)}$$

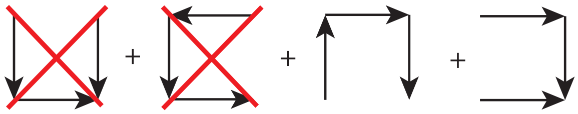

$$\frac{(\mathrm{Ez}*)}{\mathrm{Ez_{tot}}} = \frac{\qquad}{D} = \frac{k_7k_9\mathrm{E}_1k_{11} + k_9\mathrm{E}_1k_{11}k_{14}}{D} \qquad \text{(S13)}$$

and finally,

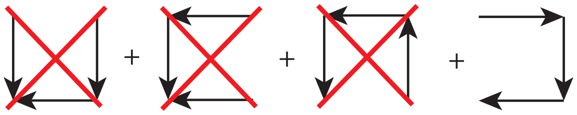

$$\frac{(\mathrm{Ez^*{\cdot}E_2})}{\mathrm{Ez_{tot}}} = \frac{\qquad}{D} = \frac{k_9\mathrm{E}_1k_{11}k_{13}\mathrm{E}_2}{D} \qquad \text{(S14)}$$

The red crosses indicate that these interconversion patterns do not contribute, as the final reaction which produces P is irreversible. It may be mentioned that an overall irreversibility of the controllers' reaction network is a necessary condition such that negative feedback loops can exhibit integral control. For more details, see end of chapter *Enzymatic mechanisms considered* in the main paper.

The denominator D is the sum of all numerators of Eqs S11, S12, S13, and S14, i.e.,

$$
\begin{aligned}
D = {} & k_{11}k_{13}\mathrm{E}_2k_7 + k_{13}\mathrm{E}_2k_7k_{10} + k_7k_{10}k_{12} + k_{14}k_{12}k_{10} \\
& + k_{13}\mathrm{E}_2k_7k_9\mathrm{E}_1 + k_7k_9\mathrm{E}_1k_{12} + k_{14}k_{12}k_9\mathrm{E}_1 \\
& + k_7k_9\mathrm{E}_1k_{11} + k_9\mathrm{E}_1k_{11}k_{14} + k_9\mathrm{E}_1k_{11}k_{13}\mathrm{E}_2
\end{aligned} \tag{S15}
$$

The steady state velocity v is then given by

$$
v = \frac{\mathrm{dP}}{\mathrm{d}t} = \left(\frac{k_7\mathrm{Ez}_{\mathrm{tot}}}{D}\right) k_9k_{11}k_{13}(\mathrm{E}_1)(\mathrm{E}_2) \tag{S16}
$$

where $\mathrm{k}_7(\mathrm{Ez}_{\mathrm{tot}})=\mathrm{V}_{\mathrm{max}}$.

References

[1] King, E. L.; Altman, C. *J Phys Chem* **1956**, *60*(10), 1375–1378.

[2] Segel, I. H. *Enzyme Kinetics: Behavior and Analysis of Rapid Equilibrium and Steady State Enzyme Systems;* Wiley: New York, 1975.
